# Oral Insulin

**DOI:** 10.1186/1758-5996-2-66

**Published:** 2010-11-08

**Authors:** Sanjay Kalra, Bharti Kalra, Navneet Agrawal

**Affiliations:** 1 Bharti Hospital, Karnal, India; 2 Diabetes, Obesity and Thyroid Centre, Gwalior, India

## Abstract

Oral insulin is an exciting area of research and development in the field of diabetology. This brief review covers the various approaches used in the development of oral insulin, and highlights some of the recent data related to novel oral insulin preparation.

## Introduction

Insulin has saved the lives of countless people, since its discovery in 1922. However, the difficulties associated with subcutaneous administration, need for frequent self monitoring of glucose, and non-physiological action profiles of insulin prompted many physicians (and their patients!) to ask for non-injectable methods of insulin delivery.

The first oral insulin preparations were tried, with poor results, by Joslin, in 1922 and 1923. Since then, many researchers have worked on this concept, with no success [1].

Currently, however, a few insulin molecules designed for oral administration are in clinical trials, and promise to reach clinical use soon. This review analyses the current status of oral insulin. It focuses only on those preparations which are actively being worked on, and does not cover methodologies on which work has stopped. Clinical data on this topic is relatively scarce, and this explains the short length of the review.

## The need for Oral Insulin

Resistance to injectable insulin has been identified as a major reason for clinical inertia and lack of achievement of target glycemic goals. Physicians as well as patients fear the complexity of insulin regimes, the risk of hypoglycemia, and the chances of weight gain, as well as the necessity of a needle prick, with insulin therapy. Insulin is perceived to have a high index of intrusion as the conventional insulins need to be given prior to meals [2].

Patients anticipate the early development of an oral insulin, as it will be easy to administer, have a lower index of intrusion, be more convenient, and have more compliance or adherence from the patient, and finally lead to better glycemic control, and thus, prevention of complications of diabetes [3].

Oral insulin may improve β-cell function by providing β-cell rest [4], and may help in preventing diabetes via induction of 'oral tolerance' or immuno modulation [5,6].

Oral insulin is able to achieve a high porto-systemic gradient, as it is delivered to the liver from the gastrointestinal tract. This reduces systemic insulin exposure and may obviate the excessive weight gain sometimes seen with subcutaneous insulin.

Oral insulin may also be able to correct the blunting of first-phase release of insulin [7], which is difficult with conventional subcutaneous insulins.

## Barriers to oral insulin

The gastro intestinal tract has many physiological barriers which prevent optimal delivery of oral insulin.

Physiological function of the gut enzymes is to break large "active" proteins into smaller "inactive" amino acids so that they can overcome the second absorption barrier "tight epithelium" in the gastro intestinal tract. These two essential barriers have been created by Mother Nature to prevent body from potentially dangerous proteins. Researchers are trying to selectively break this natural defence mechanism so that helpful large "active protein" drugs can cross this barrier and produce desired pharmacological effects.

Enzymes such as pepsin, trypsin, chymotrypsin, carboxypeptidase and pancreatin break proteins such as insulin into amino acids with great efficiency [8].

The insulin that survives faces a barrier to absorption as well. The intestine has tightly bound columnar cells with hydrophobic proteins called occludins, and a thick layer of mucin, which prevent absorption of insulin [9,10].

Further to these barriers the absorbed insulin has to go through first pass metabolism at liver before reaching at peripheral sites of action unlike sub-cutaneously administered insulin which directly reaches peripheral sites. This direct entry of insulin into liver has been postulated as being of physiological advantage by several authors however long-terms effect of this are yet to be seen [8,11].

Readers interested in more information on barriers to oral insulin delivery are referred to more detail review on the subject by Heinemann L and Jacques Y [3].

## Approaches to oral insulin

Various workers have tried different approaches to overcome the above mentioned barriers.

Insulin has been encapsulated in nanoparticles by muco-adhesive polymers such as chitosan, poly (lactic-co-glycolic acid) (PLGA), and alginate [12-17], which prevent enzymatic degradation, and allow absorption across the epithelial layer in Peyer's patches [18]. This approach depends on absorption of insulin in the colon, which is too delayed to correct first phase insulin secretion deficiency.

Insulin has also been co-administered with protease inhibitors such as bacitracin, sodium glycocholate and camostat mesilate [19], which improve the absorption rate. However, the long-term effects of this approach are not known.

Insulin can also be modified by PEGylation, or by adding polyethylene glycol, which changes the pharmacokinetics, prevents enzymatic degradation, and increases absorption [20].

Other workers have used permeation enhancers such as fatty acid salts [21], Zonula occludens toxin (ZOT) [22]^**, **^**and **monosodium N-(4-chlorosalicyloyl)-4-aminobutyrate (4-CNAB) [23]. These permeation enhancers may lead to local inflammation and encourage gastro intestinal infections.

## Recent reports of oral insulin

An insulin analogue, IN-105, delivered orally in form of tablets has been modified by linking a single short-chain amphiphilic oligomer, through a covalent non-hydrolysable amide bond, to the free amino acid group on the Lys-β29 residue of recombinant human insulin. This improves its solubility, stability and systemic absorption[24]. Solubility is increased by PEGylation, while stability may be due to steric hindrance.

IN-105 has similar insulin receptor binding and metabolic activity similar to that of human insulin, while exhibiting much lower (25-30%) insulin-like growth factor, receptor binding and mitogenic activity [24].

IN-105 has been studied in an open label, sequential ascending dose, multicentric study in 20 subjects with type 2 diabetes poorly controlled on stable metformin monotherapy, to assess the dose requirement of the molecule.

The average maximum fall in plasma glucose levels was 18.1, 26.1, 29.0 and 30.8% after administration of 10, 15, 20 and 30 mg doses. The duration of fall of glucose increased with increasing doses. Five subjects experienced six mild episodes of hypoglycemia, all of which occurred between 30 and 60 minutes of administration of varying doses (15 to 30 mg) of IN-105. Other adverse events were hypertriglyceridemia, dizziness and hyperhydrosis. It appears that study demonstrated a dose dependant decrease in post prandial glucose [24].

From the results published so far by the manufactures it appears that IN-105 is rapid acting oral insulin which could potentially find place in control of post prandial hyperglycemia. Long term effects on HbA1c reduction and beta cell function are yet to be seen. It is presently being studied in a phase 3 trial in type 2 diabetes, as well as a phase 1 trial in type 1 diabetes, and is perhaps in the most advanced development stage of all oral insulin.

In a recently published study it appears that one of the oral insulins under development did not appear to show dose-response relationship and the induced metabolic effect was very small. A single centre open label, randomized, two period cross over, isoglycemic glucose clamp study has been performed on 10 male insulin-naïve patients with type 2 diabetes who received 300 units of oral insulin and 15 units of human regular insulin on two separate days [23]. The oral insulin was combined with 400 mg of a drug carrier molecule called monosodium N-(4-chlorosalicyloyl)-4-aminobutyrate (4-CNAB), which binds non covalently to insulin, and improves gastro intestinal absorption [25,26].

Oral insulin led to early enhanced pharmacokinetic and pharmacodynamic responses with a higher and faster peak of plasma insulin concentration as compared to subcutaneous insulin. (C_max _93 ± 71 vs. 33 ± 11 μU/ml, and T_max _27 ± 9 vs.161 ± 83 min) [23].

Bioavailability was 26 ± 28%, 7 ± 4%, and 2 ± 1% relative to subcutaneous insulin during 0-1 hour, 0-2 hours and 0-6 hours. Biopotency was 55 ± 92%, 12 ± 9%, and 3 ± 1% for the same periods.

This proof-of-concept study has revealed the rapid and short action of oral insulin, albeit with high variability, and the efficacy of 4-CNAB as an absorption enhancer.

A similar randomized open label, cross over study has been performed in 16 patients with type 2 diabetes, using 150 and 300 U oral insulin (Capsulin) and 12 IU regular insulin. The hypoglycemic effect of 150 and 300 U of this oral insulin was similar to each other, and was = 50% of subcutaneous insulin[27,28]. This oral insulin is packaged in an enteric coated capsule, which dissolves in the jejunum, and contains excepients that enhance its absorption.

Another novel delivery mechanism is hepatic directed vesicles (HDV-I) loaded with insulin, which are <150 nm diameter liposomes that contain insulin attached a specific proprietary hepatocyte-targeting molecule (HTM). HDV-1 is stable at low pH, in blood, is able to avoid enzymatic degradation, and has high biopotency, exhibited by its low dose of 5 U [3].

Studies have been performed in type 1 and type 2 diabetes [29], and a larger study is underway. Metabolic control is much poorer with this oral insulin than with subcutaneous insulin [29].

Another oral insulin in development for treatment of type 1 and type 2 diabetes is ORMD-0801. The results of a single-blind, open-label, single-center, Phase IIa study in 8 T1DM, male subjects (ages 24-41, diabetics for 2-28 years, HgA1C 6.63-8.63%), regularly treated with no-peak insulin was recently presented. Two capsules of ORMD-0801 (8 mg insulin each) were orally administered to fasting subjects. A standard 400 kcal meal was served at 10, 45 or 90 min thereafter. Blood samples were collected over the 6-hr post insulin administration to monitor insulin levels. Significant increases in insulin levels were detected in 61% of the treatment sessions (T_max _40-180 min), irrespective of timing of meals. In all cases, insulin levels returned to baseline within 45-300 minutes of peak recordings, demonstrating full clearance from the bloodstream. Glucose C_max _was reached at an approximate 100-min lag from start of meal (Range: 60-150 min), which in 17/23 cases returned to basal levels before the end of the monitoring session. No serious adverse events were recorded throughout the study. It appears that this study showed some promise for control of post prandial hyperglycemia in type 1 diabetes patients [30].

The company reported brief results in May 2010 of a randomized, double-blind, placebo-controlled, multi-centered 6-week study conducted in South Africa which evaluated responses of 29 Type 2 diabetes patients to ORMD-0801. The results stated a clinically relevant reductions in insulin, c-peptide, fasting blood glucose and Hb1Ac in the ORMD-0801 cohort, when compared to the placebo. Also insulin and CRP levels were found to be statistically significant; however complete results are not yet out.

In a recent press release company developing ORMD-0801 announced successful completion of a an exploratory clinical trial testing the effectiveness of its oral insulin capsule in Type 1 diabetes patients suffering from uncontrolled diabetes, however complete results are not yet out [31].

Another oral insulin under development known as NN1952 has recently completed Phase 1 studies with an objective to study safety, tolerability, pharmacokinetics and pharmacodynamics in healthy subjects and subjects with type 1 and type 2 diabetes. This insulin has been compared to placebo and insulin aspart injection. It will be interesting to see the results of this study [23].

## The Future of oral insulin

The physiological barriers to absorption of oral insulin, its low bioavailability and low biopotency, and the high inter-patient variability, are challenges that researchers need to overcome before oral insulin can be considered as suitable candidate for treatment of diabetes mellitus.

Recent reports, including proof-of-concept studies, and early clinical studies, however, are reason for optimism that oral insulin will soon become a reality.

## Competing interests

The authors declare that they have no competing interests.

## Authors' contributions

All authors have contributed equally to the conceptualization, literature search and writing of the review. All authors have read and approved the final manuscript.
